# Age-Related Differences in Accelerometer-Assessed Physical Activity and Sleep Parameters Among Children and Adolescents With and Without Autism Spectrum Disorder: A Meta-Analysis

**DOI:** 10.1001/jamanetworkopen.2023.36129

**Published:** 2023-10-06

**Authors:** Xiao Liang, Justin A. Haegele, Sean Healy, Andy Choi-Yeung Tse, Hui Qiu, Shi Zhao, Chunxiao Li

**Affiliations:** 1 Department of Rehabilitation Sciences, The Hong Kong Polytechnic University, Hong Kong; 2 Department of Human Movement Sciences, Old Dominion University, Norfolk, Virginia; 3 Center for Movement, Health, & Disability, Old Dominion University, Norfolk, Virginia; 4 Department of Physical Education and Sports Science, University of Limerick, Limerick, Ireland; 5 Department of Health and Physical Education, The Education University of Hong Kong, Hong Kong; 6 Department of Educational Administration and Policy, The Chinese University of Hong Kong, Hong Kong; 7 JC School of Public Health and Primary Care, The Chinese University of Hong Kong, Hong Kong; 8 School of Physical Education & Sports Science, South China Normal University, Guangzhou, China; 9 Adapted Physical Activity Laboratory, South China Normal University, Guangzhou, China

## Abstract

**Importance:**

Physical inactivity and sleep disorders are health-related concerns of youth with autism spectrum disorder (ASD) that can persist from childhood and exacerbate core symptoms. However, evidence on group differences in accelerometer-assessed physical activity and sleep parameters among youth with and without ASD is inconclusive and age-specific effects remain unclear.

**Objective:**

To synthesize evidence on group differences in accelerometer-assessed physical activity and sleep parameters and examine the moderating effects of age between children and adolescents with and without ASD.

**Data Sources:**

American Psychological Association PsychInfo, CINAHL Ultimate, ERIC, MEDLINE, SPORTDiscus with Full Text, and Web of Science from inception to February 2023.

**Study Selection:**

Two independent reviewers screened articles for observational research comparing accelerometer-assessed physical activity levels and sleep parameters in children and adolescents with and without ASD.

**Data Extraction and Synthesis:**

After developing a standardized form, relevant data were extracted. Quality was assessed using the McMaster Critical Review Form and rated based on sample, measurement, and analyses. The Preferring Reporting Items for Systematic Reviews and Meta-Analyses guideline was followed.

**Main Outcomes and Measures:**

The primary outcomes were actigraphy-measured MVPA, sleep latency, sleep efficiency, total sleep time, and wake after sleep onset. Data were pooled using a random-effects model. Hedges *g* was used to express the effect size index. Meta-regression on age was also performed to investigate the potential moderating effects.

**Results:**

Collectively, 1757 studies were initially identified. Among 104 articles that were assessed, 28 were included, comprising moderate-to-vigorous physical activity (MVPA), 4 sleep parameters, and 73 independent effect sizes. A total of 28 studies were included in analysis, comprising 805 children and adolescents with ASD and 1573 without ASD (age range, 5.1-16.9 years). Compared with peers without ASD, children and adolescents with ASD had a small-to-moderate difference in MVPA (*g* = −0.450; 95% CI, −0.622 to −0.277), total sleep time (*g* = −0.332; 95% CI, −0.574 to −0.090), sleep efficiency (*g* = −0.424; 95% CI, −0.645 to −0.203), and a moderate difference in sleep latency (*g* = 0.514; 95% CI, 0.351 to 0.677) measured by actigraphy. Children and adolescents with ASD experienced an age-related decline in moderate-to-vigorous physical activity (β = −0.049 [95% CI, −0.097 to −0.001]; *P* = .045), indicating that younger children with ASD showed a smaller difference in MVPA compared with their peers without ASD. Moderating effects of age on sleep parameters were not significant.

**Conclusions and Relevance:**

The findings of this meta-analysis suggested that children and adolescents with ASD had lower MVPA and worse sleep than peers without ASD, and the difference in MVPA varied with age. These findings reinforce the need for public health initiatives aimed at reducing these group disparities.

## Key Points

**Question:**

What differences in accelerometer-assessed physical activity and sleep parameters exist in children and adolescents with and without autism spectrum disorder (ASD), and are associations affected by age?

**Findings:**

This meta-analysis including 28 studies with 805 participants found that youth with ASD were associated with lower moderate-to-vigorous physical activity (MVPA), total sleep time, and sleep efficiency but higher sleep latency than peers without ASD. An age-related decline was only observed in MVPA.

**Meaning:**

These results suggest that public health initiatives are needed to reduce disparities in MVPA and sleep health among children and adolescents with ASD.

## Teaser

This meta-analysis examines the association of autism spectrum disorder in youth with physical activity and sleep quality in studies using accelerometer measurements.

## Introduction

Autism spectrum disorder (ASD) is a neurodevelopmental condition characterized by impaired social communication, repetitive behaviors, and restricted interests^1^ affecting approximately 1% of children worldwide.^2^ Alongside the core symptoms, physical inactivity and sleep disturbances, as critical health behaviors, typically manifest in school-aged children with ASD.^2,3^ It has been proposed that sensory sensitivity abnormalities might contribute to physical inactivity and sleep problems among children with ASD.^4,5^ Sensory modulation issues among children with ASD may affect their ability to engage in physical activity with intense sensory stimulation.^6^ Similarly, children with ASD who experience hypersensitivity in response to sensory stimulation tend to be more likely to feel anxious at bedtime, resulting in difficulty falling asleep.^7^

Systematic reviews and meta-analyses of randomized clinical trials have shown that engaging in physical activity, especially in moderate-to-vigorous physical activity (MVPA), is associated with diverse physical and psychological benefits in children with ASD, such as reduced stereotypic behaviors,^8^ improved executive function,^9^ motor proficiency,^10^ and social functioning.^11^ Indeed, due to the evidence underpinning its effectiveness, exercise (ie, planned, structured, and repetitive physical activity) has been identified as an evidence-based strategy for school-aged youth with ASD.^12^ However, despite the growing evidence for the wide-ranging benefits of physical activity for youth with ASD, data indicate that this population tends to be less active and is less likely to meet the minimum World Health Organization–recommended MVPA guidelines of 60 minutes per day of MVPA,^13^ as compared with their peers without ASD.^14,15^

Sleep disorders occur in 50% to 80% of school-aged children with ASD.^16^ Common sleep problems include sleep onset delay, shorter sleep duration, early morning awakenings, and night waking.^17,18,19^ Generally, there is a consensus that children with ASD experience poorer sleep than their peers without ASD.^20,21^ However, the reported sleep problems exhibit high heterogeneity,^16^ which may be due to age differences of the samples between studies, interindividual differences across ASD groups, and variations in assessment methods (eg, parent-reported questionnaire and sleep diary vs actigraphy and polysomnography [PSG] measures).^16^

Despite physical activity and sleep health being recognized as critical health behaviors for children with ASD, there remains a lack of consensus on the differences in these behaviors among youth with ASD vs youth without ASD. We identified 4 gaps in current research that warrant a meta-analysis of group differences in physical activity and sleep among children and adolescents with and without ASD. First, a 7-country survey indicated that only 7.2% of youth with ASD met the daily recommended physical activity guidelines (MVPA over 60 minutes), while 55.9% met the sleep duration guidelines (9 to 11 hours/night).^22^ Reliance on parental reports can be problematic as they are likely to underestimate the child’s physical inactivity and disturbed sleep over time^23^ and may have difficulty removing bias from their observation, leading to inaccurate records. Second, a 2022 meta-analysis addressing accelerometer-measured physical activity^24^ indicated that children and adolescents with ASD have significantly lower physical activity levels than their peers without ASD. However, their review included 2 studies involving preschool children with ASD^25,26^ and 3 studies assessed physical activity only during segments of the school day (eg, inclusive physical education and recess),^27,28,29^ which limits interpretations relevant to habitual physical activity, which is the typical activity pattern of children in daily life. Third, device-based assessments of sleep parameters are inconsistent across studies.^21^ In particular, PSG-based studies showed several disturbed sleep parameters in youth with ASD, while actigraphy-based studies reported significantly longer sleep latency in youth with ASD than their peers without ASD, with nonsignificant results documented in other sleep parameters.^21^ Perhaps this discrepancy is attributable to the fact that their review included very few studies which reported accelerometer-assessed sleep parameters (6 studies). Finally, previous reviews have suggested that physical activity levels and sleep issues in children with ASD are age-related. Specifically, younger children displayed increased physical activity,^15^ bedtime resistance, and anxiety,^30^ while older children experienced increased insomnia.^30^ However, the moderating role of age has not been examined in a meta-analysis of accelerometer-assessed PA and sleep quality. Moreover, increasingly physical activity and sleep are being considered as part of the 24-hour movement framework and therefore being studied in unison.^31^ Reporting on meta-analyses on both behavioral outcomes in 1 report may continue to encourage this more holistic perspective of daily health behaviors. The present study, therefore, aimed to (1) systematically review and perform a meta-analysis of mean differences in physical activity levels and 4 sleep parameters (eg, sleep latency, total sleep time, sleep efficiency, wake after sleep onset [WASO]) among children and adolescents with and without ASD, as measured via accelerometers, and (2) examine the moderating effect of age.

## Methods

This study adhered to the Preferred Reporting Items for Systematic Review and Meta-analyses (PRISMA) guidelines. The protocol for this study has been registered in the International Prospective Register of Systematic Reviews (PROSPERO) (CRD42023398508).

### Search Strategy

To identify all relevant published articles that reported on the comparison of accelerometer-assessed physical activity levels or sleep parameters between children and adolescents with and without ASD, electronic searches were performed using 6 databases, from inception to February 2023: American Psychological Association PsychInfo (via Ovid), CINAHL Ultimate (via EBSCOhost), ERIC (via EBSCOhost), MEDLINE (via Ovid), SPORTDiscus with Full Text (via EBSCOhost), and Web of Science. The search was limited to articles written in English on human-related topics. A snowballing search was performed to identify additional relevant articles, where the reference lists of the retrieved studies (backward snowballing), and references that cited the eligible studies (forward snowballing) were checked for additional eligible studies.

### Eligibility Criteria

Inclusion criteria were studies that (1) compared accelerometer-assessed physical activity levels and/or sleep parameters (eg, MVPA, sleep latency, total sleep time, sleep efficiency, WASO), recorded on at least 3 valid days or nights,^32^ between children and adolescents with and without ASD; (2) were observational-based research (cross-sectional, case-control, and cohort); (3) reported the duration of MVPA, sleep latency, total sleep time, and WASO in minutes and sleep latency in percentage; (4) were peer-reviewed, English-language articles with full-text availability; (5) involved participants with ASD aged between 5 and 18 years diagnosed using standardized diagnostic tools (eg, *Diagnostic and Statistical Manual of Mental Disorders* [Third Edition] [*DSM-3*]; *DSM-4*; *DSM-5*; *International Statistical Classification of Diseases and Related Health Problems, Tenth Revision* [*ICD-10*]; and Autism Diagnostic Observation Schedule–Second Edition [ADOS-2]) or parental reports; and (6) provided complete research data whereby physical activity and sleep continuity could be computed. Exclusion criteria were studies that (1) collected data from self-reported measurements; (2) only included participants with ASD and not those without; (3) were written in a language other than English; (4) reported intervention-based research (eg, clinical and field trials); (5) were review studies, case or government reports, conference papers, book chapters, or policy documents; and (6) included preschool children (ages 0 to 4 years old) or adults (ages 18 years and older).

### Outcome Variables

MVPA refers to the length of moderate-to-vigorous physical activity levels in the form of duration in minutes per day. Sleep latency refers to the time in minutes from lights out to falling asleep. Total sleep time refers to the total time in minutes spent asleep per night. WASO refers to time awake after sleep onset, namely awake time length in minutes between sleep onset and sleep offset. Sleep efficiency refers to the percentage of total sleep time divided by the time in bed between sleep onset and sleep offset.

### Study Selection and Data Extraction

Multi-step screening was conducted by 2 independent reviewers (X.L. and H.Q.).^15^ Agreement was reached by consensus-aimed discussion. Interrater reliability was measured using Cohen κ statistic.^33^ After developing a standardized data extraction form, the relevant study characteristics were extracted: bibliographic details (author, year, and country or regions in which studies occurred), research design, participant characteristics (sample size, sex, age, medication status, and diagnosis), physical activity measures and levels, sleep measures and duration or percentages, recorded duration, and major findings.

### Quality Assessment

The methodological quality of each study was assessed by 2 reviewers (X.L. and H.Q.) using the McMaster Critical Review Form for Quantitative Studies^34^ and rated based on 3 key criteria.^35^ The sample criterion evaluated whether selection bias was reduced, whether the sample size was suitable for the research design and questions, and whether the participant characteristics were clearly described. The measurement criterion examined whether measurement bias was controlled (eg, reliability and validity of the measurement tool, recall/memory). The analyses criterion examined whether the reported analyses were appropriate for the research questions and outcome measures (eg, reported statistical significance, point estimates and variability, and discussed clinical importance).^35^ Each criterion was scored as 1 star (no evidence that the study met the requirement), 2 stars (insufficient evidence or unclear reporting), or 3 stars (the evidence met the criteria).^15,35^

### Statistical Analysis

A meta-analysis was performed using Comprehensive Meta-Analysis software version 3 (Biostat Inc). The MVPA, sleep latency, total sleep time, and WASO (in minutes) and sleep efficiency (as a percentage) were used to calculate effect size. Hedges *g* was used to measure the effect size index with small-sample bias correction.^36^ A random-effects model using the DerSimonian-Laird method was employed to compute the potentially heterogeneously distributed effect sizes with a 95% CI between the groups. The magnitude of Hedges *g* values was classified as small (under 0.2), moderate (0.5), and large (over 0.8) effect sizes.^37^ Statistical heterogeneity was assessed using *I^2^* with Cochran *Q* statistic *P* value. The *I^2^* values represented small (25% or lower), medium (50%), or large (75% or higher) heterogeneity.^38^ A cut-off point of 50% *I^2^* was set to evaluate the heterogeneity across studies, with an *I^2^* value above 50% indicating the existence of heterogeneity. Sensitivity analysis was used to determine whether the elimination of any study influenced the overall effect size.^11^ Funnel plots and Egger regression were used to assess possible publication bias. Finally, meta-regression analysis was performed to compare the physical activity levels and sleep parameters of those with and without ASD depending on age (mean age). Statistical significance was considered *P* < .05 for all tests.

## Results

### Study Identification

In total, 1757 articles were identified upon initial screening (eFigure 1 in Supplement 1). After removing duplicates, 677 articles were screened by title or abstract. A total of 104 abstracts and 25 articles met the inclusion criteria (κ = 0.86 and κ = 0.92, respectively). Three articles, identified via a manual snowball search, also met the inclusion criteria. Finally, 28 studies^39,40,41,42,43,44,45,46,47,48,49,50,51,52,53,54,55,56,57,58,59,60,61,62,63,64,65,66^ were selected for systematic review and meta-analysis as the data was considered sufficient.

### Characteristics of Included Studies

The 28 studies included 805 participants with ASD (679 male [84.3%]) and 1573 without ASD (895 male [56.9%]); ages ranged from 5.1 to 16.9 years (eTable 1 in Supplement 1). Only 9 studies^39,40,42,44,45,54,58,64^ (32.1%) reported medication use status, of which, 5 studies^42,44,45,58,64^ included medicine-naive children and adolescents with ASD as participants. While 24 studies^39,40,41,42,44,45,46,47,48,49,50,51,52,53,55,56,57,59,60,62,63,64,65,66^ (85.7%) confirmed ASD diagnosis using standardized diagnostic methods (eg, *DSM-4*, *DSM-5*, *ICD-10*, and ADOS-2), only 7 studies^39,41,49,50,53,54,57^ (25.0%) provided a clear classification of ASD severity. The differences in physical activity levels and sleep parameters between children and adolescents with and without ASD are summarized in the Table. Twelve studies^43,46,47,49,50,53,55,57,60,61,62,65^ recorded physical activity levels of the duration of the actigraph measurements ranging from 4 to 14 days and measured MVPA duration as an outcome to represent daily physical activity. Furthermore, we identified within-group differences in physical activity levels between weekdays^47,49,50,55,60,65^ and weekend days^55,60,65^ among participants with ASD and found that children and adolescents with ASD spent more time in MVPA on weekdays (67.56 min/d) than on weekend days (39.01 min/d). Furthermore, 17 studies^39,40,41,42,44,45,47,48,51,52,54,56,58,59,63,64,66^ assessed sleep parameters via actigraphy, across 3 to 7 nights of measurement. Lastly, 7 studies^44,45,47,51,52,53,56^ (25.0%) controlled for confounders such as sex and age.

**Table.** Summary of Included Studies on Moderate-to-Vigorous Physical Activity (MVPA) and Sleep Parameters

Study name, year (country)	Study design	Measurement model	Duration of actigraph data	Accelerometer-assessed MVPA or sleep parameters			Major findings^a^	Confounders
				Parameters	ASD, mean (SD)	TD, mean (SD)		
Allik et al,^39^ 2006 (Sweden)	Cross-sectional	Actiwatch	7 d	SL (min); weekdays	32.2 (17.9)	15.7 (10.6)	SL positive	NA
				SL (min); weekend	21.5 (20.0)	11.4 (10.3)	SL positive	
				TST (min); weekday	511 (34.7)	523 (35.0)	NS	
				TST (min); weekend	514 (44.4)	522 (42.5)	NS	
				SE (%); weekdays	87.1 (3.6)	90.3 (4.1)	NS	
				SE (%); weekend	88.6 (4.7)	90.1 (4.1)	NS	
Allik et al,^40^ 2008 (Sweden)	Longitudinal	Actiwatch	7 d	SL (min); weekdays	25.3 (22.1)	17.8 (11.3)	NS	NA
				SL (min); weekend	28.1 (25.1)	14.2 (16.3)	NS	
				TST (min); weekend	434 (32.5)	445 (39.5)	SE positive for weekends	
				TST (min); weekend	462 (50.8)	465 (44.3)	NS	
				SE (%); weekdays	81.8 (5.0)	83.5 (4.0)	NS	
				SE (%); weekend	79.5 (5.6)	83.4 (5.2)	NS	
Baker et al,^51^ 2013 (Australia)	Cross-sectional	MicroMini Motionlogger	7 d	SL (min)	59.30 (41.27)	33.83 (16.14)	SL positive	NA
				TST (min)	470.95 (57.82)	492.01 (40.11)	NS	
Bandini et al,^60^ 2013 (US)	Cross-sectional	Actical	7 d	MVPA (min); weekdays	48.0	59.2	NS	Children without ASD are more likely to be an only child
				MVPA (min); weekend	53.5	52.1	NS	
Bennett et al,^61^ 2022 (US)	Cross-sectional	ActiGraph GT3x	4 d	MVPA (min)	29.0 (15.4)	38.0 (18.9)	NS	NA
Bricout et al,^62^ 2018 (France)	Cross-sectional	SenseWear Pro-3	7 d	MVPA (min)	27 (29)	42 (24)	NS	NA
Chua et al,^63^ 2022 (UK)	Cross-sectional	MotionWatch8	5 nights	SL (min); Singapore cohort	30.15 (17.41)	20.35 (13.50)	SL positive	School session
				SL (min); UK cohort	60.00 (48.55)	21.15 (13.23)	SL positive	
				TST (min); Singapore cohort	420 (71)	392 (34)	NS	
				TST (min); UK cohort	455 (61)	487 (34)	NS	
				SE (%); Singapore cohort	74.23 (10.03)	78.15 (6.87)	NS	
				SE (%); UK cohort	76.49 (9.86)	82.01 (4.83)	SE positive	
Goldman et al,^64^ 2009 (US)	Cross-sectional	AW-64 Actiwatch	7 d	SL (min)	53.4 (25.6)	33.7 (33.2)	SL positive	NA
				TST (min)	481.6 (56.8)	475.9 (38.8)	NS	
				SE (%)	80.9 (6.6)	84.7 (4.6)	SE positive	
				WASO (min)	39.5 (12.6)	31.7 (12.4)	NS	
Haegele et al,^65^ 2021 (US)	Cross-sectional	ActiGraph GT3x	7 d	MVPA (min); weekend	36.52 (18.28)	37.00 (14.80)	NS	NA
				MVPA (min); weekend	27.20 (22.04)	35.63 (45.63)	NS	
Hering et al,^66^ 1999 (Israel)	Cross-sectional	Ambulatory actigraph	3 nights	SL (min)	22.59 (1.02)	23.26 (1.23)	NS	NA
				TST (min)	425.8 (47.7)	431.8 (80.2)	NS	
				SE (%)	88.71 (4.45)	87.19 (5.25)	NS	
Jeon et al,^41^ 2023 (UK)	Cross-sectional	MotionWatch8	7 d	SL (min); Korean cohort	25.38 (17.84)	19.3 (11.7)	NS	Age
				SL (min); UK cohort	42.09 (39.51)	25.3 (14.6)	SL positive	
				TST (min); Korean cohort	432.6 (42.0)	439.8 (43.8)	NS	
				TST (min); UK cohort	479.4 (52.2)	495.0 (36.6)	NS	
				SE (%); Korean cohort	78.93 (5.79)	78.0 (5.0)	NS	
				SE (%); UK cohort	78.98 (8.05)	81.3 (3.87)	NS	
Kosaka et al,^42^ 2021 (Japan)	Cross-sectional	Actiwatch	7 d	SL (min)	4.5 (4.7)	4.0 (2.7)	NS	NA
				TST (min)	439.0 (37.8)	460.3 (28.4)	NS	
				SE (%)	82.8 (4.8)	86.6 (3.1)	SE positive	
				WASO (min)	70.2 (17.9)	57.1 (16.1)	WASO positive	
Lobenius-Palmér et al,^43^ 2018 (Sweden)	Cross-sectional	ActiGraph, GT1M	7 d	MVPA (min)	79 (63)	142 (80)	MVPA positive	NA
Martinez-Cayuelas et al,^44^ 2021 (Spain)	Cross-sectional	Ambulatory circadian monitoring	7 d	SL (min)	26.5 (26.9)	14.4 (13.75)	SL positive	NA
				TST (min)	517.3 (50.6)	543.7 (31.6)	NS	
				SE (%)	88.20 (5.62)	91.85 (3.09)	SE positive	
				WASO (min)	24.5 (9.78)	23.02 (5.18)	NS	
Martinez-Cayuelas et al,^45^ 2022 (Spain)	Cross-sectional	Ambulatory circadian monitoring	7 d	SL (min)	21.16 (15.58)	11.60 (7.8)	SL positive	NA
				TST (min)	510.88 (46.90)	546.63 (26.9)	TST positive	
				SE (%)	89.36 (4.48)	92.30 (1.90)	SE positive	
Moludi et al,^46^ 2019 (Iran)	Cross-sectional	ActiGraph GT3x	7 d	MVPA (min)	28.68 (18.94)	33.70 (19.97)	NS	NA
									Mughal et al,^59^ 2020 (UK)	Cross-sectional	Actiwatch 8	7 d	SL (min)	38.18 (34.12)	26.08 (26.49)	NS	Socioeconomic status, sex, age
TST (min)	444.33 (63.03)	486.55 (4.44)	TST positive														
SE (%)	72.68 (7.55)	80.02 (6.99)	SE positive														
Nguyen et al,^47^ 2021 (France)	Cross-sectional	Sensewear Pro Armband3	5 d	MVPA (min); weekdays	156 (75)	209 (59)	MVPA positive	NA
				SL (min)	13.6 (9.7)	15.9 (6.6)	NS	
				TST (min)	420 (54)	426 (47)	NS	
				SE (%)	74.9 (7.1)	74.4 (7.4)	NS	
				WASO (min)	113.0 (41.0)	126.7 (49.3)	NS	
Pace et al,^48^ 2016 (France)	Cross-sectional	Sensewear Pro Armband3	7 nights	TST (min)	413.8 (37.2)	412.0 (46.6)	NS	NA
				SE (%)	70 (7)	69 (8)	NS	
Pan et al,^49^ 2015 (Taiwan)	Cross-sectional	ActiGraph GT1M	5 d	MVPA (min); weekdays	69.61 (50.30)	97.07 (47.67)	MVPA positive	NA
									Pan et al,^50^ 2016 (Taiwan)	Cross-sectional	ActiGraph GT1M	7 d	MVPA (min); weekdays	64.23 (48.78)	90.68 (49.14)	MVPA positive	NA
MVPA (min); weekend	63.14 (84.00)	55.78 (36.09)	NS														
Phung and Goldberg,^52^ 2017 (US)	Cross-sectional	Micro motionlogger	7 d	SL (min)	42.48 (27.40)	22.46 (11.56)	SL positive	Sex
				TST (min)	419.35 (84.61)	378.61 (70.77)	TST positive	
				SE (%)	91.92 (6.63)	88.49 (8.93)	NS	
Sandt and Frey,^53^ 2005 (US)	Cross-sectional	ActiGraph MT1	14 d	MVPA (min)	127.5 (72.3)	162.1 (45.6)	MVPA positive	Sex
Souders et al,^54^ 2009 (US)	Cross-sectional	MicroMini-Motionlogger	7 nights	SL (min)	34.42 (21.94)	21.71 (8.97)	SL positive	NA
				TST (min)	452.00 (65.07)	469.57 (52.63)	NS	
				SE (%)	83.63 (7.93)	84.21 (6.23)	NS	
				WASO (min)	88.03 (41.8)	87.46 (32.99)	NS	
Stanish et al,^55^ 2017 (US)	Cross-sectional	Actical	7 d	MVPA (min); weekdays	31.0	55.2	MVPA positive	Sex, age
				MVPA (min); weekend	12.2	40.9	MVPA positive	
Surtees et al,^56^ 2019 (UK)	Cross-sectional	Actiwatch 2	7 d	SL (min)	38 (32)	29 (26)	NS	NA
				TST (min)	485 (39)	492 (45)	NS	
				SE (%)	82.08 (4.17)	83.88 (5.68)	NS	
				WASO (min)	52 (17)	48 (16)	NS	
Thomas et al,^57^ 2022 (Australia)	Cross-sectional	ActiGraph GT3x	8 d	MVPA (min)	87.03 (38.28)	82.10 (40.45)	NS	NA
									Tse et al,^58^ 2020 (Hong Kong)	Cross-sectional	ActiGraph GT3x	7 d	TST (min); weekdays	462 (30)	498 (36)	TST positive (weekdays and weekend)	NA
TST (min); weekend	492 (36)	558 (30)	SE positive (weekdays and weekend)														
SE (%); weekdays	81.1 (6.4)	90.7 (8.3)	WASO positive (weekdays and weekend)														
SE (%); weekend	86.3 (9.1)	94.8 (9.7)	NS														
WASO (min); weekdays	60.0 (27.2)	40.5 (26.9)	NS														
WASO (min); weekend	51.6 (36.3)	21.9 (33.5)	NS														

Abbreviations: MVPA, moderate-to-vigorous physical activity; NA, not applicable; NS, not significant; SE, sleep efficiency; SL, sleep latency; TST, total sleep time; WASO, wake after sleep onset.

^a^ Statistically significant associations are reported as positive or negative associations.

### Meta-Analysis of Accelerometer-Assessed Physical Activity Levels

**Figure 1.**
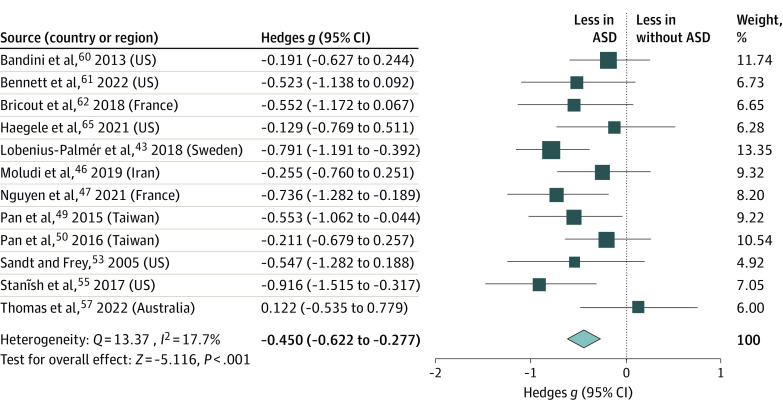
Meta-Analysis of Accelerometer-Assessed Moderate-to-Vigorous Physical Activity ASD indicates autism spectrum disorder. Size of the boxes vary by study weighting.

A significant small-to-moderate group difference in MVPA was observed in children and adolescents with ASD compared with those without (κ = 12; *g* = −0.450 [95% CI, −0.622 to −0.277]), with small heterogeneity (*Q* = 13.370; *I*^2^ = 17.7%; *P* < .001) (Figure 1). Overall, children and adolescents with ASD undertook less daily MVPA (mean [SD], 58.73 [39.09] min/d) than peers without ASD (77.03 [52.03] min/d). Sensitivity analysis revealed no significant changes in the effect size following the elimination of any individual paper (*g* = −0.450 [95% CI, −0.622 to −0.277]). The Egger test failed to find evidence for publication bias. Meta-regression analysis demonstrated that age was a significant moderator for the group difference in MVPA between children and adolescents with and without ASD (β = −0.049 [95% CI, −0.097 to −0.001]; *P* = .045) (eFigure 4 in Supplement 1), indicating that the size of the difference in physical activity levels between children and adolescents with and without ASD increased with age.

### Meta-Analysis of Accelerometer-Assessed Sleep Parameters

Of the 17 studies, 2 reported cross-cultural comparison in sleep patterns between countries: UK vs Korea,^41^ UK vs Singapore.^63^ Thus, we included contrasts between participants with and without ASD in each country and treated them as independent contrasts in the analysis. In total, 61 contrasts across 17 studies were identified: 17 effect sizes^39,40,41,42,44,45,47,51,52,54,56,59,63,64,66^ reported sleep latency results, 19 effect sizes^39,40,41,42,44,45,47,48,51,52,54,56,58,59,63,64,66^ reported total sleep time, 18 effect sizes^39,40,41,42,44,45,47,48,52,54,56,58,59,63,64,66^ reported sleep efficiency, and 7 effect sizes^42,44,47,54,56,58,64^ reported WASO.

**Figure 2.**
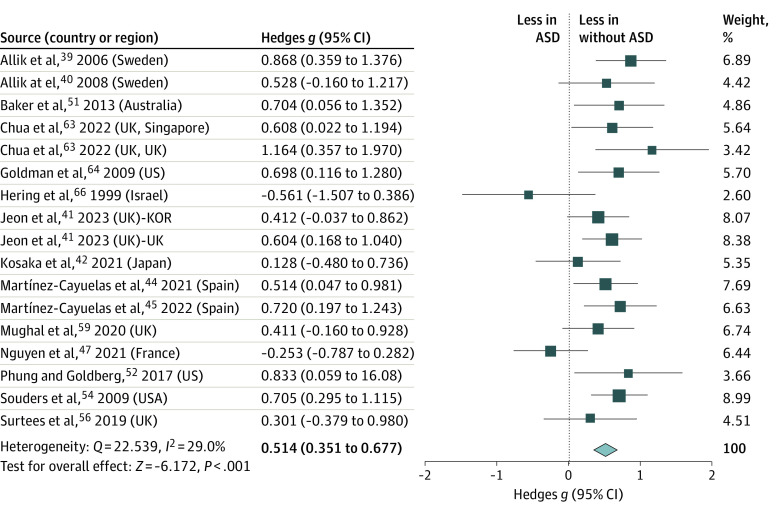
Meta-Analysis of Accelerometer-Assessed Sleep Latency ASD indicates autism spectrum disorder. Size of the boxes vary by study weighting.

A significant moderate-to-large group difference in sleep latency was observed in those with ASD compared with those without (κ = 17; *g* = 0.514 [95% CI, 0.351–0.677]), with small heterogeneity (*Q* = 22.539; *I*^2^ = 29%; *P* < .001) (Figure 2). Children and adolescents with ASD showed longer sleep latency duration (mean [SD], 32.57 [14.57] min) than their peers without ASD (20.06 [7.65] min). Sensitivity analysis revealed no significant changes in the effect size following the elimination of any individual paper. The Egger test did not find evidence for publication bias. Meta-regression analysis indicated that the group difference in sleep latency between children and adolescents with and without ASD was not moderated by age (β = 0.025 [95% CI, −0.036 to 0.885]; *P* = .42) (eTable 2 in Supplement 1).

**Figure 3.**
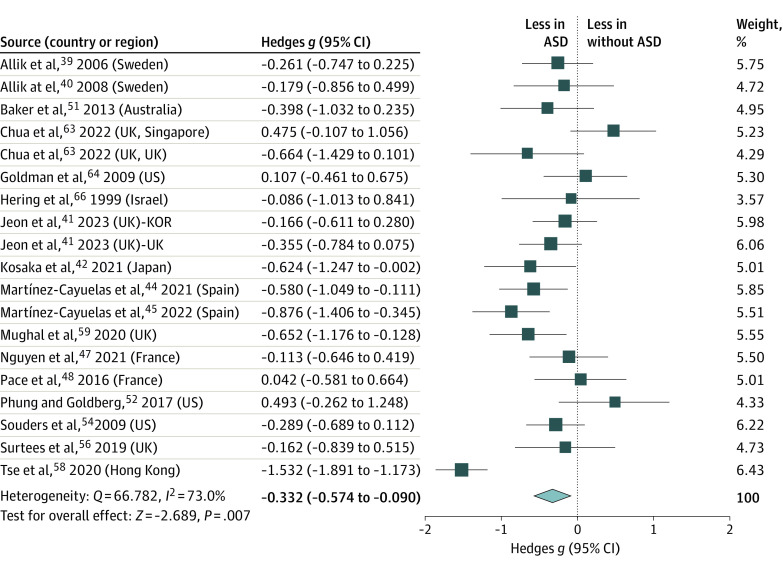
Meta-Analysis of Accelerometer-Assessed Total Sleep Time ASD indicates autism spectrum disorder. Size of the boxes vary by study weighting.

There was a statistically significant between-group differences in total sleep time (κ = 19; *g* = −0.332 [95% CI, −0.574 to −0.090]), with medium-to-large heterogeneity (*Q* = 66.782; *I^2^* = 73%; *P* = .007) (Figure 3). Total sleep time was significantly lower in children and adolescents with ASD (mean [SD], 461.00 [34.05] min) than in those without (474.54 [48.76] min). Sensitivity analysis revealed no significant changes in the effect size following the elimination of any individual paper. Egger regression revealed publication bias among the included papers; 5 additional studies were needed to balance the total sleep time plot (eFigure 2 in Supplement 1). Meta-regression analysis demonstrated that age did not moderate the group differences in total sleep time between children and adolescents with and without ASD (β = 0.037 [95% CI, −0.054 to 0.128]; *P* = .43) (eTable 2 in Supplement 1).

**Figure 4.**
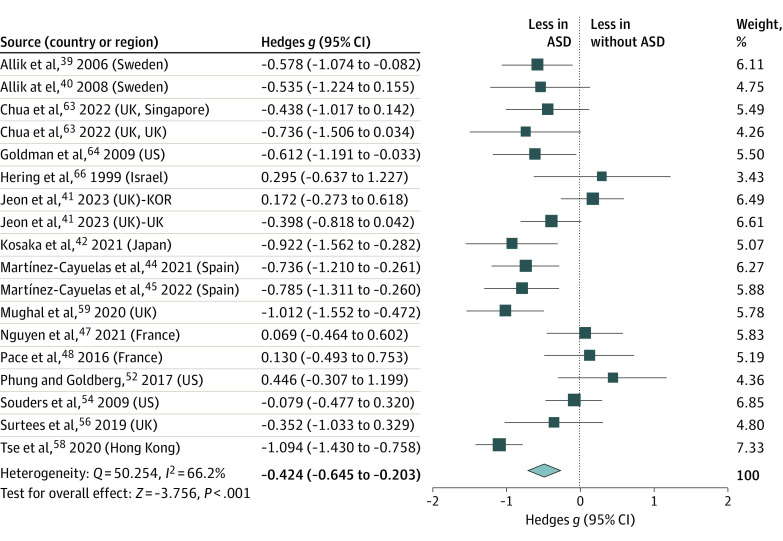
Meta-Analysis of Accelerometer-Assessed Sleep Efficiency ASD indicates autism spectrum disorder. Size of the boxes vary by study weighting.

A statistically significant group difference in sleep efficiency was observed (κ = 18; *g* = −0.424 [95% CI, −0.645 to −0.203]) with medium heterogeneity (*Q* = 50.254; *I*^2^ = 66%; *P* < .001) (Figure 4). Significantly lower SE was found in youth with ASD (mean [SD] 81.82% [6.02%]) than in peers without ASD (84.52% [6.39%]). Sensitivity analysis revealed no significant changes in the effect size following the removal of any individual paper. The Egger test did not identify publication bias. Meta-regression analysis demonstrated nonsignificant differences in sleep efficiency between children and adolescents with and without ASD depending on age (β = 0.060 [95% CI, −0.033 to 0.153]; *P* = .209) (eTable 2 in Supplement 1).

Between-group differences in WASO were not statistically significant (κ = 7; *g* = 0.324 [95% CI, −0.002 to 0.650]), with medium heterogeneity (*Q* = 18.466; *I^2^* = 68%; *P* = .051) (eFigure 3 in Supplement 1). Descriptively, WASO was slightly longer in children with ASD (mean [SD] 62.34 [27.95] min) than in those without (54.55 [36.09] min). As less than 10 studies were included in the analysis (7 studies total), analyses for publication bias and meta-regression were not conducted.

### Quality Assessment

Six studies^39,41,42,45,46,51^ met all 3 criteria for quality assessment (eTable 1 in Supplement 1). All studies used a convenience sample; male participants dominated sample selection. Only 7 studies^39,41,42,45,46,49,51^ provided a detailed classification of ASD severity. Only 1 study^65^ reported actigraphy-based simultaneous physical activity and sleep measurements, with most studies focusing on 1 health indicator independently. All studies addressed the research questions, reported statistical significance, and discussed clinical importance.

## Discussion

This study explored between-group differences in accelerometer-assessed physical activity levels and sleep parameters between children and adolescents with and without ASD. Youth with ASD had less MVPA, longer sleep latency, shorter total sleep time, and lower sleep efficiency compared with those without ASD. No differences were observed in WASO between groups. Notably, differences in MVPA varied with age, but this was not the case for sleep parameters.

Consistent with previous reviews,^14,15^ our findings showed that accelerometer-assessed habitual physical activity was significantly lower in children and adolescents with ASD than in their peers without ASD. Moreover, those with ASD were less likely to meet the World Health Organization–recommended daily MVPA.^13^ Consistent with previous reviews,^15,24^ our findings confirmed that children and adolescents with ASD (aged 5.5 to 15.9 years) experienced age-related reductions in MVPA. A recent longitudinal study exploring physical activity behavior change timings (aged 9 to 18 years) found that MVPA disparities between children with and without ASD began at 9 years of age and progressively worsened until 13 years.^67^ Similarly, a 2020 meta-analysis^68^ indicated an annual MVPA decline of 6 minutes per day in 9-year-old male (−7.8%) and female (−10.2%) children without ASD. By adolescence, a more severe decline in MVPA exists for children with ASD. A plausible mechanism for these observed MVPA differences could be that because of early abnormal sensory sensitivity to stimuli, children with ASD demonstrate difficulties in participating in activities with physical demands and social communication as they age.^69^ Children and adolescents with ASD are less likely to participate in physical activity with higher social demands (eg, basketball, football, and volleyball), and are therefore less likely to engage in MVPA than their peers without ASD. Indeed, physical inactivity is a global pandemic, contributing to a range of chronic diseases and premature deaths.^70^ Thus, multilevel coordinated efforts at the individual, community, and governance levels are needed to increase physical activity across all ages and abilities, including among youth with ASD.^15^

Findings from accelerometer-assessed sleep parameters suggest that, on average, children and adolescents with ASD had longer sleep latency, shorter total sleep time, and lower sleep efficiency than their peers without ASD. These results were inconsistent with the findings of a previous meta-analysis,^21^ which reported that children with ASD had increased sleep onset latency as compared with children without ASD, but there were no differences in total sleep time and sleep efficiency between groups as measured by actigraphy. Notably, their review included a limited number of actigraphy-measured studies reporting sleep latency, total sleep time, and sleep efficiency, suggesting that conclusions should be interpreted with caution. Contrastingly, we provide meta-analytic evidence for the accelerometer-assessed sleep parameters,^21^ with actigraphy recordings displaying considerable sleep impairments in children and adolescents with ASD. Additionally, our results are generally consistent with those of PSG-based studies,^21^ specifically of studies that showed that, compared with children without ASD, children with ASD experienced multiple significant sleep impairments, including lower total sleep time, longer sleep latency, higher stage 1 sleep duration, and lower sleep efficiency.^21^ Usually, PSG is recorded over 1 to 2 nights in a sleep laboratory, whereas actigraphy is recorded for 7 consecutive days in the home environment. Although PSG is widely considered the criterion standard for measuring sleep architecture,^20^ the sleep laboratory may lead to worsening sleep for children with ASD, who are particularly sensitive to transfer from a familiar environment. Hence, actigraphy offers more naturalistic sleep measurements in real-world settings. Additionally, discrepancies between sleep parameters measurements were noted, whereby PSG-assessed total sleep time (standardized mean difference, −0.90) and sleep efficiency (−1.20) in a previous study^21^ significantly differed with current actigraphy-assessed total sleep time (*g* = −0.332) and sleep efficiency (*g* = −0.424); thus, both PSG and actigraphy should be implemented to ensure accurate measurements.

The recently developed 24-hour Movement Behavior Framework recommends at least 60 minutes of MVPA per day, as well as 540 to 660 minutes of sleep hours per night, for school-aged children and adolescents.^71^ Unfortunately, studies included in this analysis showed that children and adolescents with ASD tend not to adhere to the recommended guidelines. It has been shown that the specific combinations of movement behaviors (eg, high MVPA and long sleep time) are associated with lower odds ratios for developing physiological health issues, such as overweight and obesity^72^ and unfavorable body mass index scores,^22^ among children with ASD. Thus, rather than consider MVPA and sleep separately, future research focused on youth with ASD should consider the intercorrelated associations between physical activity and sleep, as well as screen-time, within the 24-hour movement behavior framework.

In the current study, differences between those with and without ASD in sleep parameters did not vary with age, which is inconsistent with findings of another meta-analysis that revealed age-related differences in SL and SE between children with and without ASD (aged 2 to 19 years).^20^ However, their review only included 10 studies and combined the results of actigraphy and PSG, causing potential selection and insensitive measurement bias. Moreover, in parent-reported studies of children with ASD aged 6 to 11 years, parasomnia and bedtime resistance appeared to decline with age.^73,74^ Such variability may be explained by age and measurement differences between studies. In the future, larger PSG- or actigraphy-recorded studies of children and adolescents with and without ASD will be pivotal to gaining new insights into group differences in sleep parameters from childhood to adolescence.

### Limitations

This study had several limitations. First, the number of available studies measuring WASO was limited, which may have caused underestimation or overestimation of WASO effect sizes. Second, male participants dominated the ASD group (84.3%), which restricted our ability to detect sex differences in physical activity and sleep parameters. Third, physical activity and sleep actigraphy recording protocols were inconsistent across studies, which may distort the synthesis of results owing to high heterogeneity. Fourth, only 9 studies (32%) reported medication status, causing difficulties in interpreting their effects on physical activity and sleep quality. Fifth, only 25% of the included studies controlled for confounders such as sex and age. As such, sensitivity analyses could not be conducted on each of these confounders. Lastly, only 1 included study^47^ assessed both (physical activity and sleep) behaviors. Thus, we cannot compare the results in 1 assessed behavior and both behaviors to explore the potential confounding factors.

## Conclusion

Based on meta-analyses of actigraphy-derived estimates of physical activity and sleep parameters, this review found small-to-moderate differences, in favor of children and adolescents without ASD, in MVPA, total sleep time, and sleep efficiency. A moderate-to-large difference, in favor of children and adolescents without ASD was demonstrated for sleep latency. Importantly, an age-related MVPA decline was observed in our study. These findings reinforce the need for public health initiatives aimed at reducing disparities in physical activity and sleep health among children and adolescents with ASD. Moreover, the findings signal the need for a standard protocol for actigraphy measurement in children and adolescents with ASD.

## Notes

Supplement 1. **eFigure 1.** PRISMA Flow Diagram of the Selection of Studies **eFigure 2.** Funnel Plot for Visual Inspection of Publication Bias of Total Sleep Time **eFigure 3.** Meta-Analysis of Accelerometer-Assessed WASO **eFigure 4.** Meta-Regression of MVPA Between Children and Adolescents With and Without ASD Depending on Age **eTable 1.** Summary of Participants’ Characteristics and Quality Assessment of Included Studies (ASD vs Without ASD) **eTable 2.** Moderator Analysis of Group Difference in MVPA and Sleep Parameters Between Children and Adolescents With and Without ASD

Supplement 2.
Data Sharing Statement
