# Supplementary material for: Age-Related Differences in Accelerometer-Assessed Physical Activity and Sleep Parameters Among Children and Adolescents With and Without Autism Spectrum Disorder: A Meta-Analysis
Source: JAMA Netw Open. 2023 Oct 6;6(10):e2336129. doi: 10.1001/jamanetworkopen.2023.36129 (PMC10559179; doi:10.1001/jamanetworkopen.2023.36129)
Supplement: Supplement 1. — eFigure 1. PRISMA Flow Diagram of the Selection of Studies eFigure 2. Funnel Plot for Visual Inspection of Publication Bias of Total Sleep Time eFigure 3. Meta-Analysis of Accelerometer-Assessed WASO eFigure 4. Meta-Regression of MVPA Between Children and Adolescents With and Without ASD Depending on Age eTable 1. Summary of Participants’ Characteristics and Quality Assessment of Included Studies (ASD vs Without ASD) eTable 2. Moderator Analysis of Group Difference in MVPA and Sleep Parameters Between Children and Adolescents With and Without ASD [file jamanetwopen-e2336129-s001.pdf]

Supplemental Online Content

Liang X, Haegele JA, Healy S, et al. Age-related differences in accelerometer-assessed physical activity and sleep parameters among children and adolescents with and without autism spectrum disorder: a meta-analysis. *JAMA Netw Open.* 2023;6(10):e2336129. doi:10.1001/jamanetworkopen.2023.36129

eFigure 1. PRISMA Flow Diagram of the Selection of Studies

eFigure 2. Funnel Plot for Visual Inspection of Publication Bias of Total Sleep Time

eFigure 3. Meta-Analysis of Accelerometer-Assessed WASO

eFigure 4. Meta-Regression of MVPA Between Children and Adolescents With and Without ASD Depending on Age

eTable 1. Summary of Participants' Characteristics and Quality Assessment of Included Studies (ASD vs Without ASD)

eTable 2. Moderator Analysis of Group Difference in MVPA and Sleep Parameters Between Children and Adolescents With and Without ASD

This supplemental material has been provided by the authors to give readers additional information about their work.

© 2023 Liang X et al. *JAMA Network Open.*

eFigure 1. PRISMA flow diagram of the selection of studies

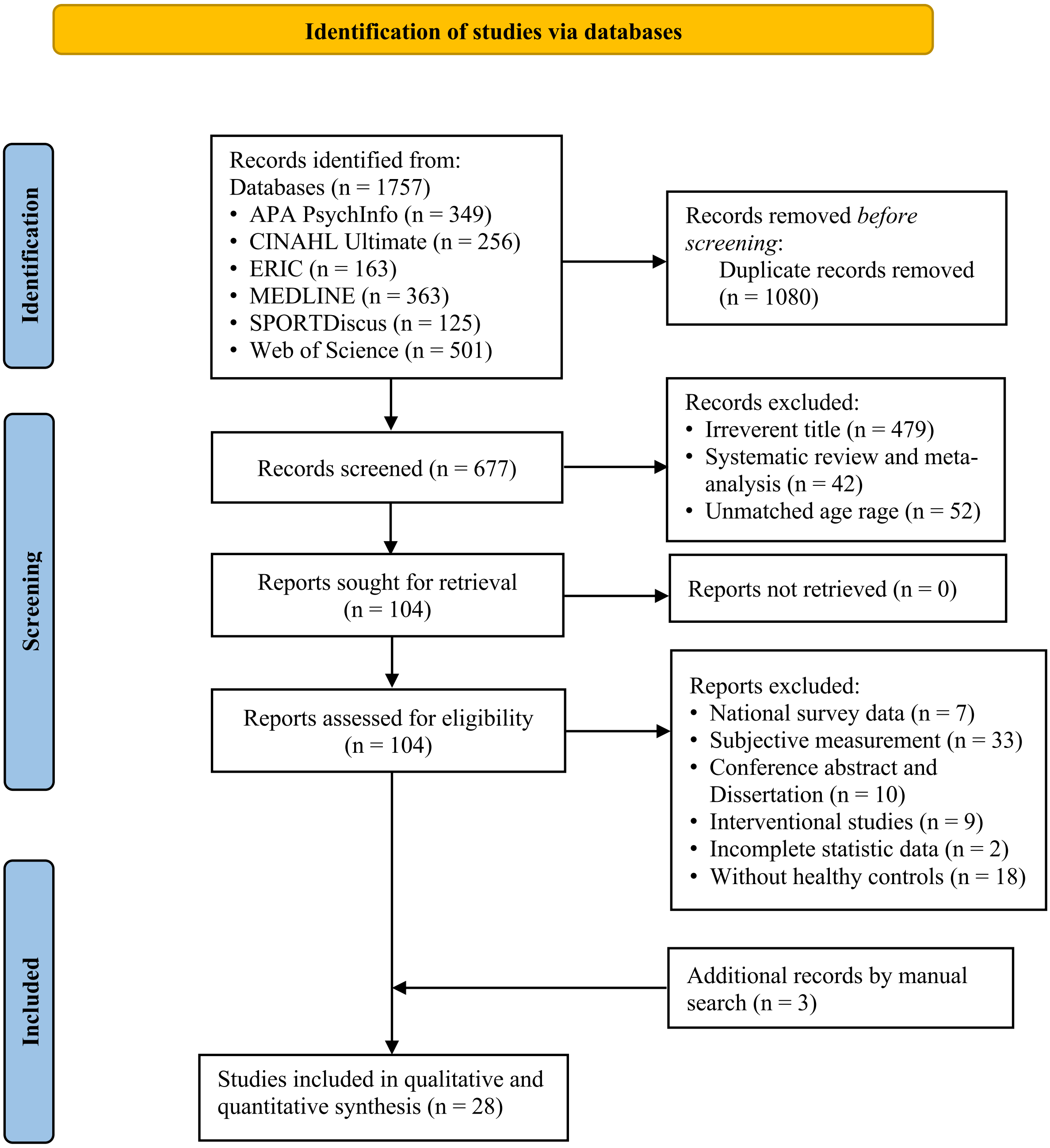

eFigure 2. Funnel plot for visual inspection of publication bias of total sleep time

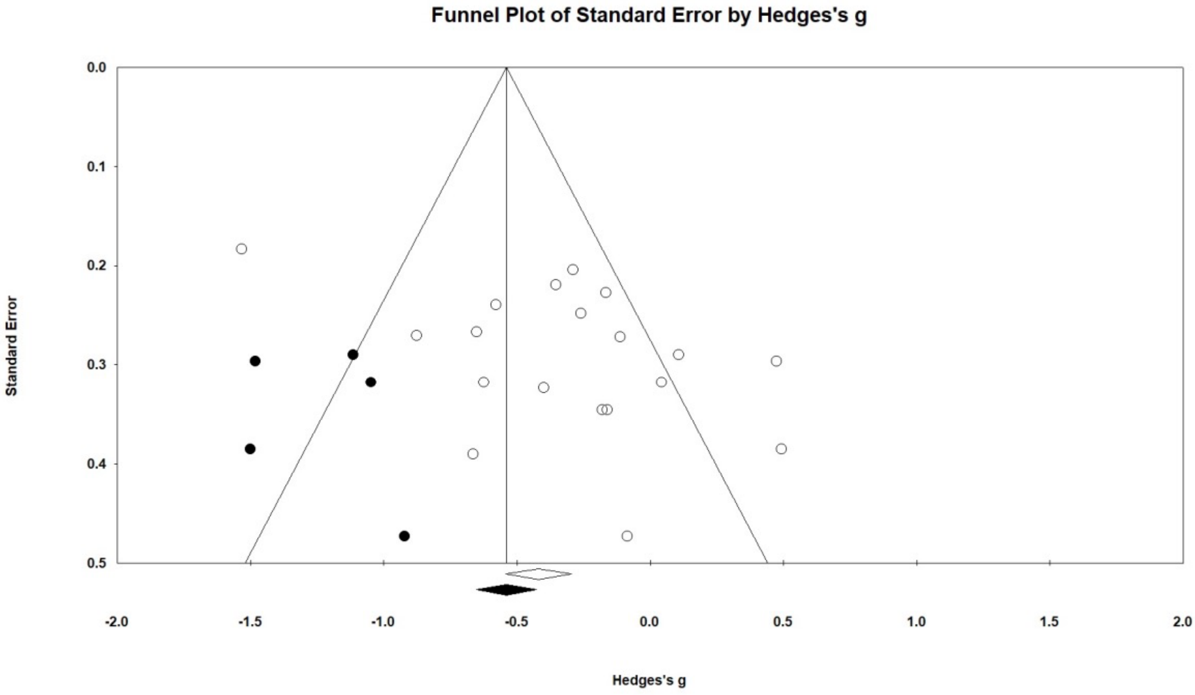

eFigure 3. Meta-analysis of accelerometer-assessed WASO

Source (Country/Region)	Hedges's *g* (95% CI)	Weight
Goldman et al,[64] 2009 (USA)	0.613 (0.034, 1.192)	12.98%
Kosaka et al,[42] 2021 (Japan)	0.754 (0.125, 1.384)	12.07%
Mart¡nez-Cayuelas et al,[44] 2021 (Spain)	0.172 (-0.289, 0.633)	15.27%
Nguyen et al,[47] 2021 (France)	-0.313 (-0.848, 0.222)	13.80%
Souders et al,[54] 2009 (USA)	0.015 (-0.384, 0.413)	16.55%
Surtees et al,[56] 2019 (UK)	0.236 (-0.442, 0.914)	11.26%
Tse et al,[58] 2020 (Hong Kong)	0.782 (0.458, 1.106)	18.06%
Heterogeneity: Q=18.466, I^2=67.5%<br>*Test for overall effect*: Z=1.950, p=0.051	**0.324 (-0.002, 0.650)**	**100%**

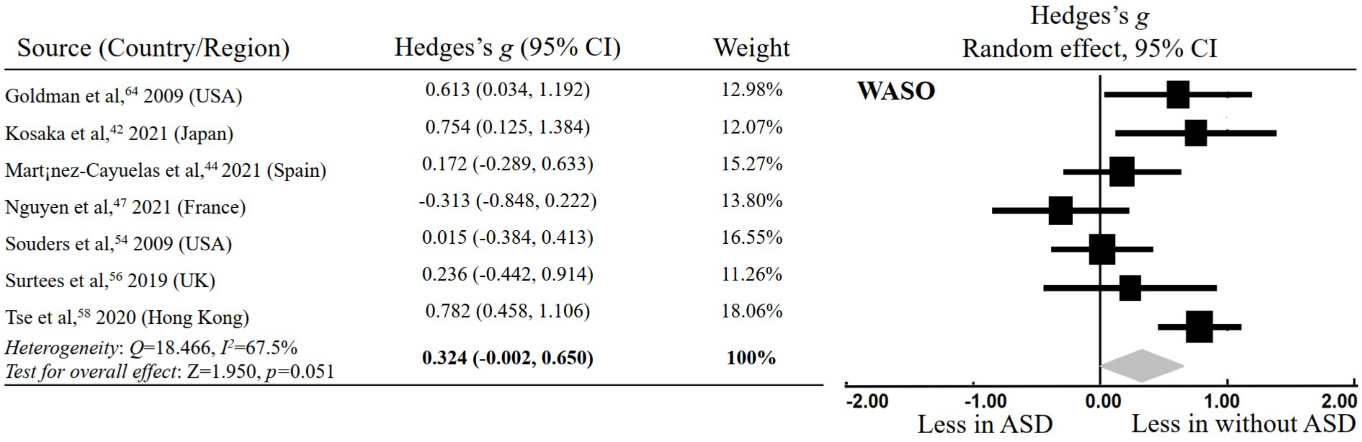

eFigure 4. Meta-regression of MVPA between children and adolescents with and without ASD depending on age

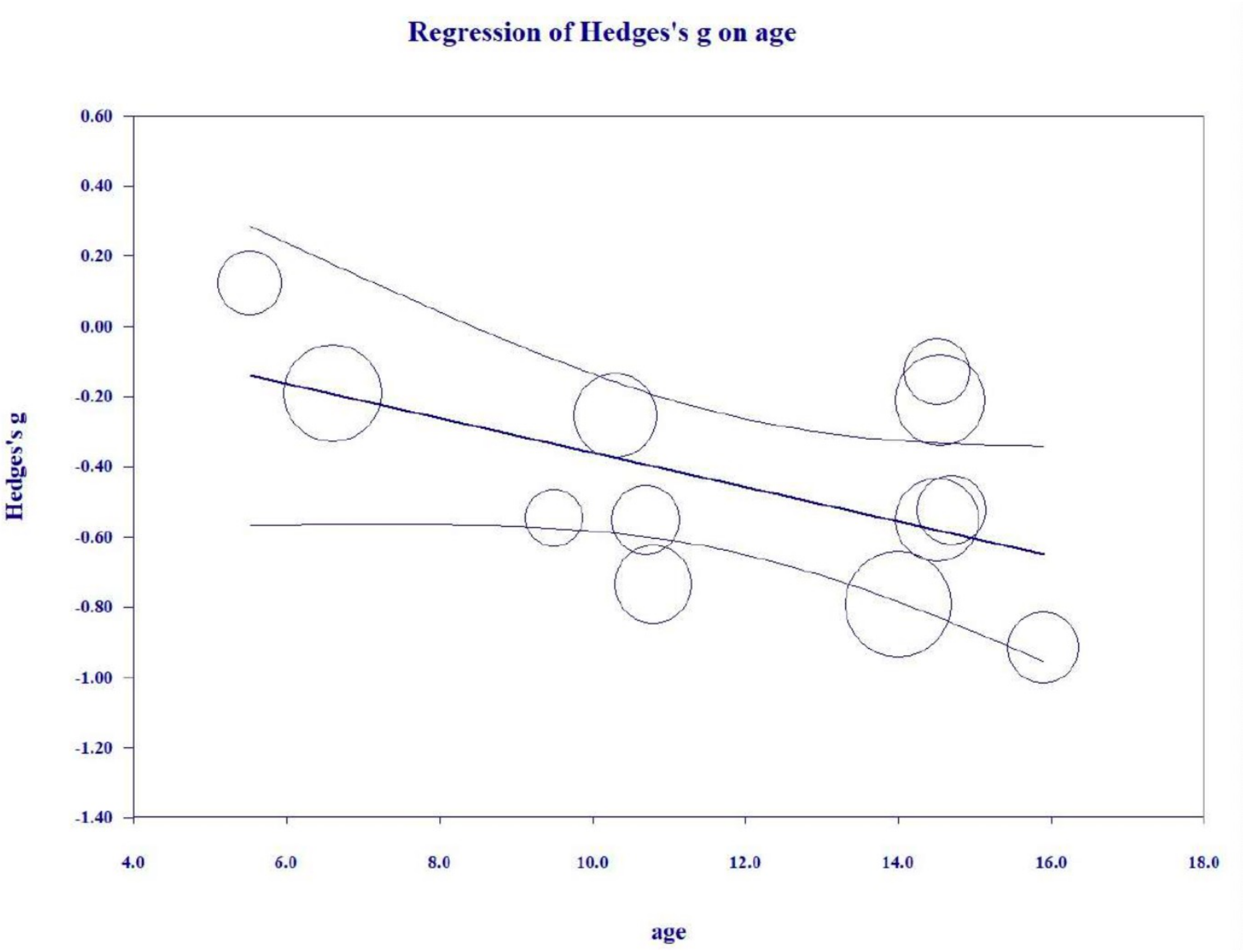

eTable 1. Summary of participants' characteristics and quality assessment of included studies (ASD Vs without ASD)

Study Name (Year, Country/ Region)	Sample size (ASD vs without ASD)	Age (M±SD)	Sex (Male%) (ASD vs without ASD)	Medication use# (ASD)	Diagnostic Methods (Classification of ASD Severity)	Quality Criteria		
						Sample	Methods	Analysis
Allik et al,[39] 2006 (Sweden)	• 32 ASD<br>• 32 without ASD	• 10.8±1.20<br>• 10.9±1.30	• M-87.5%<br>• M-87.5%	Yes	ICD-10<br>(Asperger (19); High-functioning Autism (13))	***	***	***
Allik et al,[40] 2008 (Sweden)	• 16 ASD<br>• 16 without ASD	• 13.7 (11.7-15.5)<br>• 13.7 (11.5-15.6)	• M-87.5%<br>• M-87.5%	Yes	ICD-10	**	***	***
Baker et al,[51] 2013 (Australia)	• 15 ASD<br>• 25 without ASD	• 15.5±1.3<br>• 15.5±1.1	• M-81.5%<br>• M-81.5%	Yes	Social Communication Questionnaire	*	**	**
Bandini et al,[60] 2013 (USA)	• 35 ASD<br>• 47 without ASD	• 6.6±2.1<br>• 6.7±2.4	• M-83%<br>• M-78%	N/A	Autism Diagnostic Interview-Revised	**	***	***
Bennett et al,[61] 2022 (USA)	• 25 ASD<br>• 17 without ASD	• 14.7±1.5<br>• 14.5±1.5	• M-70.6%<br>• M-70.6%	N/A	Parent reports	*	**	**
Bricout et al,[62] 2018 (France)	• 20 ASD<br>• 20 without ASD	• 10.7±1.2<br>• 10.0±1.6	• M-100%<br>• M-100%	N/A	DSM-5 & Autism Diagnostic Observation Schedule	**	**	**
Chua et al,[63] 2022 (UK)	• 37 ASD (26 SGP & 11 UK)<br>• 36 without ASD (20 SGP & 16 UK)	• 9.74±1.73<br>• 9.04±1.70	N/A	N/A	DSM-5	*	**	**
Goldman et al,[64] 2009 (USA)	• 42 ASD<br>• 16 without ASD	• 5.85±2.0<br>• 6.90±1.9	• M-90%<br>• M-75%	None	Autism Diagnostic Observation Schedule	**	***	***
Haegele et al,[65] 2021 (USA)	• 18 ASD<br>• 18 without ASD	• 14.51±1.54<br>• 14.44±1.38	• M-27.8%<br>• M-27.8%	N/A	Parent reports	*	**	**
Hering et al,[66] 1999 (Israel)	• 8 ASD<br>• 8 without ASD	• 8.0±3.0<br>• 8.0±2.3	• M-87.5%<br>• M-87.5%	N/A	DSM-4	*	**	**
Jeon et al,[41] 2023 (UK)	• 68 ASD (33 KOR & 35 UK)<br>• 96 without ASD (45 KOR & 51 UK)	• 8.27±1.89 KOR-A<br>• 9.14±1.97 KOR-T<br>• 9.21±1.97 UK-A<br>• 8.75±1.87 UK-T	• M-78.8% KOR-A<br>• M-51.1% KOR-T<br>• M-74.3% UK-A<br>• M-54.9% UK-T	N/A	Childhood Autism Rating Scale-2<br>((High-functioning (30); Standard (38))	***	***	***
Kosaka et al,[42] 2021 (Japan)	• 20 ASD<br>• 20 without ASD	• 5.1±0.9<br>• 5.2±1.3	• M-85%<br>• M-60%	None	DSM-5	**	***	***
Lobenius-Palmér et al,[43] 2018 (Sweden)	• 25 ASD<br>• 800 without ASD	• 14.0±3.7<br>• 11.8±3.1	• M-76%<br>• M-44.5%	N/A	Parent reports	*	**	**

Martinez-Cayuelas et al,[44] 2021 (Spain)	• 52 ASD<br>• 27 without ASD	• 9.85±3.07<br>• 8.81±2.14	• M-90.4%<br>• M-74%	None	DSM-5 & Autism Diagnostic Observation Schedule	**	***	***
Martinez-Cayuelas et al,[45] 2022 (Spain)	• 37 ASD<br>• 24 without ASD	• 9.40±2.6<br>• 8.42±2.4	• M-91.9%<br>• M-75%	None	Autism Diagnostic Observation Schedule	**	***	***
Moludi et al,[46] 2019 (Iran)	• 30 ASD<br>• 29 without ASD	• 10.30±2.37<br>• 9.83±1.97	• M-100%<br>• M-100%	N/A	DSM	*	**	**
Mughal et al,[59] 2020 (UK)	• 21 ASD<br>• 45 without ASD	• 8.42±1.81<br>• 8.12±1.29	• M-81%<br>• M-51%	N/A	Childhood Autism Rating Scale	*	**	**
Nguyen et al,[47] 2021 (France)	• 50 ASD<br>• 18 without ASD	• 10.8±2.6<br>• 10.1±2.2	• M-100%<br>• M-100%	N/A	DSM-5 & Autism Diagnostic Observation Schedule	**	***	***
Pace et al,[48] 2016 (France)	• 19 ASD<br>• 19 without ASD	• 10.7±1.2<br>• 9.9±1.6	N/A	N/A	DSM-5	*	**	**
Pan et al,[49] 2015 (Taiwan)	• 30 ASD<br>• 30 without ASD	• 14.51±1.54<br>• 14.72±1.54	• M-100%<br>• M-100%	N/A	DSM-4<br>(Asperger (7); Mild (23))	***	***	***
Pan et al,[50] 2016 (Taiwan)	• 35 ASD<br>• 35 without ASD	• 14.55±1.54<br>• 14.81±1.55	• M-100%<br>• M-100%	N/A	DSM-4<br>(Asperger (10); Mild (25))	***	***	***
Phung and Goldberg,[52] 2017 (USA)	• 19 ASD<br>• 10 without ASD	• 16.88±2.50<br>• 15.73±2.00	• M-84.2%<br>• M-60%	N/A	Autism Diagnostic Observation Schedule	**	**	***
Sandt and Frey,[53] 2005 (USA)	• 15 ASD<br>• 13 without ASD	• 9.5±1.9<br>• 8.9±2.0	• M-66.7%<br>• M-61.5%	N/A	DSM-4<br>(Asperger (2); Standard (9); PDDNOS (4))	***	***	***
Souders et al,[54] 2009 (USA)	• 59 ASD<br>• 40 without ASD	• 7.53±1.92<br>• 7.09±2.09	• M-81.4%<br>• M-65%	Yes	Autism Diagnostic Observation Schedule<br>(Asperger (12); Standard (26); PDDNOS (21))	***	***	***
Stanish et al,[55] 2017 (USA)	• 16 ASD<br>• 39 without ASD	• 15.9±1.7<br>• 15.3±1.5	• M-83%<br>• M-60%	N/A	Autistic Diagnostic Interview, Revised	**	***	***
Surtees et al,[56] 2019 (UK)	• 16 ASD<br>• 16 without ASD	• 9.8<br>• 9.5	• M-62.5%<br>• M-62.5%	N/A	Autism Diagnostic Observation Schedule	**	***	***
Thomas et al,[57] 2022 (Australia)	• 17 ASD<br>• 17 without ASD	• 5.52±0.85<br>• 5.71±0.52	• M-76.5%<br>• M-47.1%	N/A	DSM-5<br>(Asperger (1); Standard (11); PDDNOS (5))	***	**	***
Tse et al,[58] 2020 (Hong Kong)	• 78 ASD<br>• 78 without ASD	• 10.05±1.08<br>• 10.05±1.08	• M-79.5%<br>• M-79.5%	None	DSM-5	**	***	***

Note: *=no criteria were met within that component; **=only some criteria were met within the component; ***=all criteria were met within that component. #: the criteria for medication use is whether the participants with a history of psychotropic or currently used drugs, including non-stimulant medications for ASD; or with a drug-naïve before study.
CRS-R:L: Conners' Rating Scale-Revised Long Version; DSM-4 and-5: Diagnostic and Statistical Manual of Mental Disorders, Fourth Edition and Fifth Edition; K-SADS-PL: Schedule for Affective Disorders and Schizophrenia for School-age Children-Present and Lifetime Version; P-ChIPS: Children's Interview for Psychiatric Syndromes;

eTable 2. Moderator analysis of group difference in MVPA and sleep parameters between children and adolescents with and without ASD

Parameters	Model	Number of contrasts (k)	*β*	SE	95% CI	Z-value	P-value
MVPA	Intercept	12	0.129	0.301	[-0.460, 0.718]	0.43	0.668
	Age	12	**-0.049***	0.024	[-0.097, -0.001]	-2.00	**0.045***
Sleep latency	Intercept	17	0.267	0.314	[-0.348, 0.885]	0.85	0.393
	Age	17	0.025	0.032	[-0.036, 0.087]	0.81	0.419
Total Sleep Time	Intercept	19	-0.694	0.475	[-1.626, 0.237]	-1.46	0.144
	Age	19	0.037	0.047	[-0.054, 0.128]	0.79	0.428
Sleep Efficiency	Intercept	18	-0.994	0.468	[-1.910, -0.077]	-2.13	0.034
	Age	18	0.060	0.048	[-0.033, 0.153]	1.26	0.209

Note: The number of contrasts means the number of comparisons included in the meta-regression.
