# Supplementary material for: Age-Related Differences in Accelerometer-Assessed Physical Activity and Sleep Parameters Among Children and Adolescents With and Without Autism Spectrum Disorder: A Meta-Analysis
Source: JAMA Netw Open. 2023 Oct 6;6(10):e2336129. doi: 10.1001/jamanetworkopen.2023.36129 (PMC10559179; doi:10.1001/jamanetworkopen.2023.36129)
Supplement: Supplement 2. — Data Sharing Statement [file jamanetwopen-e2336129-s002.pdf]

Data Sharing Statement

Liang. Age-Related Differences In Accelerometer-Assessed Physical Activity and Sleep Parameters Among Children and Adolescents With and Without Autism Spectrum Disorder. *JAMA Netw Open*. Published October 06, 2023. doi:10.1001/jamanetworkopen.2023.36129

Data
Data available: No
